# Ohio College of Dental Surgery

**Published:** 1852-07

**Authors:** 


*Ohio College of Dental Surgery.*—We are happy to learn from the Eighth Annual Announcement of the Ohio College of Dental Surgery, which we have just received ; that, at no period since the organization of the Institution, have the faculty had so much cause for encouragement, and that many of the difficulties with which it has hitherto had to contend have been overcome.

The lectures will commence the first Monday in November, and close the 20th of February.

